# Cell Therapy as Target Therapy against Colon Cancer Stem Cells

**DOI:** 10.3390/ijms24098163

**Published:** 2023-05-03

**Authors:** Elsa N. Garza Treviño, Adriana G. Quiroz Reyes, Juan Antonio Rojas Murillo, David A de la Garza Kalife, Paulina Delgado Gonzalez, Jose F. Islas, Ana Esther Estrada Rodriguez, Carlos A. Gonzalez Villarreal

**Affiliations:** 1 Laboratorio de Terapia Celular, Departamento de Bioquímica y Medicina Molecular, Facultad de Medicina, Universidad Autónoma de Nuevo León, Av. Dr. José Eleuterio González 235, Monterrey 64460, Nuevo León, Mexico; elsa.garzatr@uanl.edu.mx (E.N.G.T.); guadalupe.quirozrys@uanl.edu.mx (A.G.Q.R.); juan.rojasmrll@uanl.edu.mx (J.A.R.M.); david.delagarzaka@uanl.edu.mx (D.A.d.l.G.K.); paulina.delgadogn@uanl.edu.mx (P.D.G.); jislas.me0117@uanl.edu.mx (J.F.I.); 2 Departamento de Ciencias Básicas, Vicerrectoría de Ciencias de la Salud, Universidad de Monterrey, Ignacio Morones Prieto 4500. Jesus M. Garza, San Pedro Garza García 66238, Nuevo León, Mexico; ana.estradar@udem.edu

**Keywords:** cancer, cancer stem cells, colon cancer, cell therapy, T cells

## Abstract

Cancer stem cells (CSCs) are a small subpopulation of cells within tumors with properties, such as self-renewal, differentiation, and tumorigenicity. CSCs have been proposed as a plausible therapeutic target as they are responsible for tumor recurrence, metastasis, and conventional therapy resistance. Selectively targeting CSCs is a promising strategy to eliminate the propagation of tumor cells and impair overall tumor development. Recent research shows that several immune cells play a crucial role in regulating tumor cell proliferation by regulating different CSC maintenance or proliferation pathways. There have been great advances in cellular immunotherapy using T cells, natural killer (NK) cells, macrophages, or stem cells for the selective targeting of tumor cells or CSCs in colorectal cancer (CRC). This review summarizes the CRC molecular profiles that may benefit from said therapy and the main vehicles used in cell therapy against CSCs. We also discuss the challenges, limitations, and advantages of combining conventional and/or current targeted treatments in the late stages of CRC.

## 1. Introduction

Cancer malignancy relates to tumor heterogeneity, and it has been suggested that it is driven by a minor subpopulation of cells called cancer stem cells (CSC) [1,2]. CSCs are a small subpopulation of cancer cells that can self-renew, differentiate, and start tumor growth, thereby mediating drug resistance and cancer progression, resulting in supporting tumor recurrence and distant metastasis [3,4,5]. Furthermore, CSCs can differentiate into different cell populations with a high plasticity potential, they have a high resistance to stressful conditions in the tumor microenvironment (TME), they can induce cell death by chemotherapeutic agents [6], and have the ability to invade healthy tissues. All of these characteristics are related to tumor progression, metastasis, and resistance to antitumoral therapies [1,7].

Surgery, radiotherapy, and chemotherapy are conventional treatments. Surgery can successfully remove cancer masses from the body, while combining radiotherapy with chemotherapy can effectively lead to better results for treating numerous types of cancer [7]. Nevertheless, even when conventional cancer treatments target the bulk of the tumor, they cannot eliminate CSCs responsible for metastasis and tumor recurrence. Therapeutic alternatives to chemotherapy and surgery, such as cell therapy against CSCs, have yielded results in recent years. CSCs’ surface markers are well characterized, making possible the creation of bio-targeted therapies.

Cell therapy is a promising option in bio-directed cell-targeted therapies against CSCs, particularly with modified cells that enhance the interactions and mechanisms related to the eradication of CSCs. These cellular therapies against CSCs work in different ways: (i) they can release factors that modulate and regulate the TME and enhance the suppression of cancer cells [8] through the activation or inhibition of some signaling pathways; (ii) they can act as a cell-targeted delivery system for anticancer drugs [9], and (iii) they can induce cell apoptosis through the receptor-binging union.

In this review, we analyze the different cell types, such as T cells, NK cells, macrophages, and stem cells, based on current immunotherapy approaches, compare their advantages and disadvantages, and evaluate the main targeting against CSCs in clinical trials in advanced CRC.

## 2. Specific Targets Used in CRC Cell Therapy with Potential Antitumoral Effect against CSCs

**Figure 1:**
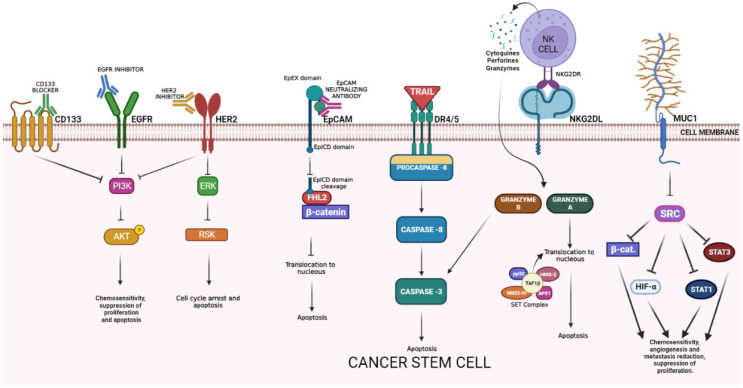
Cancer stem cell surface markers and their antitumoral mechanisms. EGFR = epidermic growth factor receptor; PI3K = Phosphatidylinositol 3 kinase; AKT = protein kinase B; HER2 = human epidermal growth factor receptor 2; ERK = extracellular signal-regulated kinases; RSK = ribosomal S6 kinase; EpCAM = epithelial cell adhesion molecule; EpEX = EpCAM extracellular domain; EpICD = EpCAM intracellular domain; FHL2 = four and a half LIM domains protein 2; DR4/5 = death receptor 4/5; TAF1 BETA = TATA-box binding protein associated factor 1; NKG2R = natural killer group 2 receptor; NKG2L = natural killer group 2 ligand; MUC-1 = mucin 1; HIF-α = hypoxia-inducible factor; STAT1 = signal transducer and activator of transcription 1.

There are specific targets in CSCs that can be used as therapeutic targets, such as CD44, CD133, CD24, and ALDH; however, these markers are not frequently used in cell therapy because they are commonly expressed in normal stem cells. Among other disadvantages, they only represent a low percentage within the tumor and have variable expression during the disease stages and after chemotherapy or radiotherapy. CRC targets have been proposed as an attractive cell therapy strategy and stand out due to overexpression in tumor tissues compared to healthy tissue, and recently to target pathways or the activity of CSCs (Figure 1). Some of the most prominent are:

**EGFR** is a transmembrane glycoprotein belonging to the protein kinase superfamily activated by ligand binding, that causes receptor dimerization and activates multiple pathways that leads to gene activation for cell survival, proliferation, and differentiation. It is also related to functions of metabolism and affects autophagy. Some reports suggest that it can sensitize CSCs to apoptosis by chemotherapy (5FU) after treatment with EGFR monoclonal antibody. [10] An in vivo model showed that anti-EGFR therapy can affect CSC numbers and counteract the chemotherapy-induced CSCs’ expansion [11]. EGFR inhibitors suppressed proliferation and induced apoptosis of CSCs by inhibiting autophosphorylation of EGFR and downstream signaling proteins, such as Akt kinase and extracellular signal-regulated kinase 1/2 (ERK 1/2) (Figure 1) [12].

**HER2** is a non/ligand-binding member of the EGFR family. It homodimerizes or heterodimerizes with other EGFR family members (HER1/EGFR, HER3, HER4), inducing the transphosphorylation of the intracytoplasmic tyrosine kinase domain and activation of a variety of downstream signal transduction pathways (e.g., RAS/RAF/ERK, PIK3K/AKT/mTOR, JAK/STAT3). The overexpression of HER2 causes hyperactivation of mitogenic signals, resulting in uncontrolled cell proliferation and cancer. *HER2* amplification and protein overexpression can be identified in approximately 6% of CRC patients [13,14,15,16,17]; both HER-2 and HER-3 are overexpressed in liver metastasis in CRC patients (8% and 75%, respectively) [18] HER2 amplification most commonly occurs in the rectum and has been linked to the resistance of EGFR-targeted therapy and lower overall survival compared to HER2 wild-type CRC [19,20]. Early onset research in the area determined that the expression of the markers CD24, CD44, ALDH, and regulated epithelial-mesenchymal transition (EMT) is seen in the presence of HER2 blockage in breast cancer [21]. HER2-overexpressing gastric cancer cells exhibited increased stemness and invasiveness and were regulated by Wnt/β-catenin signaling [22].

The epithelial cell adhesion molecule (**EpCAM**) is a type I transmembrane glycoprotein expressed mostly in the basolateral membrane of normal epithelial cells [23]. Under normal conditions, EpCAM is involved in cell-cell adhesion and the regulation of differentiation in progenitor and embryonic stem cells; however, in the context of cancer, EpCAM overexpression is related to increased cell proliferation, migration, invasion, and tumor metastasis [23,24]. Although it has been shown to be overexpressed in a wide variety of epithelial tumors, EpCAM seems to be associated with a poor prognosis in certain cancer types (colorectal, breast, prostate, gallbladder, ovarian, bladder, pancreas, and adenoid cystic carcinomas). EpCAM has been reported as a marker of better prognosis in other tumors (esophageal, renal, gastric, endometrial, thyroid, and head-and-neck carcinomas) [25]. In CRC, EpCAM is overexpressed in over 90% of all cancer cells [24]. The EpCAM neutralizing antibody exhibited antitumor effects via inhibiting the nuclear translocation of EpICD/β-catenin complexes and induced apoptosis in colon cancer cells (Figure 1) [26]. EpCAM is expressed in both healthy and cancer tissues; however, it is usually overexpressed in cancerous tumors derived from epithelial tissues [26]. Based on this fact, immunotherapies have been developed using monoclonal antibodies against EpCAM. To increase the efficacy and specificity of EpCAM treatment, combinatorial approaches have been developed, where the EpCAM antibody is fused with another anticancer molecule that targets a different cancer receptor/ligand, and improves the specificity for cancer cells, such as catumaxomab (Removab), which combines anti-CD33 antibody and anti-EpCAM antibody, and increases the specificity of the therapy [26,27]. Lastly, most therapies are locally infused to reduce the damage to healthy tissue.

Mucin 1 (**MUC-1**) is a heterodimeric transmembrane glycoprotein. Its function is related primarily with the formation of a physical barrier to lubricate and protect normal epithelial tissues and mediate signal transduction [26]. It is upregulated in response to inflammation, and is aberrantly overexpressed in diverse types of cancer [27,28]. The MUC1 molecule consists of an N-terminal subunit (MUC1-N) and C-terminal transmembrane subunit (MUC1-C) [29]. MUC1 can act as a cell surface antigen to colorectal CSC [30] resistance and angiogenesis of tumor development. Recent studies have supported a previously unreported function for MUC1-C in activating PRC2 and PRC1 in cancer cells. In the regulation of PRC2, MUC1-C drives the transcription of the EZH2 gene, binds directly to EZH2, and enhances the occupancy of EZH2 on target gene promoters with an increase in H3K27 trimethylation. Regarding PRC1, which is recruited to PRC2 sites in the hierarchical model, MUC1-C induces BMI1 transcription, forms a complex with BMI1, and promotes H2A ubiquitylation. MUC1-C thereby contributes to the integration of the PRC2 and PRC1-mediated repression of tumor suppressor genes, such as CDH1, CDKN2A, PTEN, and BRCA1. Just as PRC2 and PRC1, MUC1-C is associated with the EMT program, CSC state, and acquisition of anticancer drug resistance. In concert with these observations, targeting MUC1-C downregulates EZH2 and BMI1, inhibits EMT and the CSC state, and reverses drug resistance. These findings emphasize the significance of MUC1-C as a therapeutic target for inhibiting the aberrant PRC function and reprogramming the epigenome in human cancers [29,31].

The natural killer group 2, member D (**NKG2D**) is a stimulatory receptor found in some cytotoxic immune cells that recognizes the NKG2D ligands (NKG2DL) [7]. These molecules are not expressed in normal cells, but are upregulated in stressed and many malignant cells. In humans, two distinct categories of NKG2DL are expressed, one family consists of the major histocompatibility complex (MHC) class I polypeptide-related sequence A and B (MICA and MICB, respectively), while the other family of NKG2D ligands consists of a six-member glycoprotein family of UL16-binding proteins (ULBP1–6) [8]. In normal conditions, CSCs exhibit a lower expression of MHC-I type NKG2DL and a higher expression of UL16-binding proteins NKG2DL [9], but when there is DNA damage or cell cycle alterations, some MHC-I type NKG2DL becomes upregulated and expressed into CSCs [8]. NKG2D ligands are recognized by NKG2D receptors on subsets of neighboring cytotoxic immune cells (natural killer (NK) cells, natural killer T (NKT) cells, subsets of gamma delta (γδT cells). The natural killer group 2D (NKG2D) has recently emerged as a major activating receptor on T lymphocytes and natural killer cells. In both humans and mice, multiple genes encode ligands for NKG2D, and these ligands are non-classical MHC Class I. The NKG2D-ligand interaction triggers and activates a signal in the cell expressing NKG2D, and this promotes cytotoxic lysis of the cell expressing the ligand. Most normal tissues do not express ligands for NKG2D, but ligand expression has been documented in tumor and virus-infected cells, leading to the lysis of these cells. The tight regulation of ligand expression is important. If there is inappropriate expression in normal tissues, this will favor autoimmune processes, whilst failure to upregulate the ligands in pathological conditions would improve cancer development or the dissemination of intracellular infection [32].

**TRAIL** is a type II transmembrane protein denominated tumor necrosis factor (TNF)-related apoptosis-inducing ligand (TRAIL, CD253). It has been widely studied as a strategy for tumor elimination since cancer cells overexpress TRAIL death receptors, selectively inducing apoptosis [33]. In contrast with most chemotherapeutic drugs, this protein triggers the extrinsic apoptotic pathway in malignant cells in a p53-independent manner. TRAIL binds to death receptors DR4 (TRAIL-R1) and DR5 (TRAIL-R2), decoy receptors DcR1 and DcR2, and osteoprotegerin (OPG). The binding to its DR4 and DR5 receptors activates the homotypic DD-dependent recruitment of Fas-associated protein with death domain (FADD). This protein bridges pro-caspases 8 and 10 to form a death-inducing signaling complex (DISC), that activates caspases-8, 3, and 7, leading to apoptosis. In vitro, TRAIL can induce apoptosis in a wide variety of tumor cells. One of the main limitations of the recombinant protein TRAIL application is fast the clearance from the serum [34]. Moreover, it was observed that soluble TRAIL and monoclonal antibodies against DR4 and DR5 present potent antitumor activities in in vivo models without systemic toxicity [33]. Recombinant TRAIL treatment has been evaluated in clinical trials. Although it has had an important antitumor effect, it has a short half-life and repeated applications are needed to obtain the desired effect or systems that facilitate its continuous expression [35].

**CD133**, the cluster of differentiation 133 or prominin-1, is a five-transmembrane glycoprotein expressed in several progenitor and stem cells. It participates in the organization of the topology of the plasma membrane [36]. Researchers have proposed CD133 as a cell surface marker of CSCs since its expression seems to relate to chemoresistance, an elevated risk of distant metastasis, and relapse [36,37]. Moreover, it participates in primordial cell differentiation and EMT [38,39,40]. CSCs display an EMT phenotype, possess elevated levels of the transcription factors SNAIL and TWIST, the mesenchymal markers vimentin and fibronectin, and low levels of epithelial protein E-cadherin [41]. CD133+ CRC cells manifest CSC-like properties, such as higher levels of the SC markers OCT4 and SOX2, a tumor sphere-forming ability, and are more tumorigenic in NOD/SCID mice [41]. This finding is consistent with OCT4 and SOX2 overexpression in poorly differentiated human tumors [42]. CD133+ CSCs might escape immune surveillance by expressing the inhibitory molecule B7H1. Moreover, the innate immune system can effectively be recruited to kill CSCs using bispecific antibodies targeting CD133 [43]. CD133 can upregulate the expression of the FLICE-like inhibitory protein (FLIP) in CD133-positive cells, inhibiting apoptosis. In addition, CD133 can increase angiogenesis by activating the Wnt signaling pathway and increasing the expression of vascular endothelial growth factor-A (VEGF-A) and interleukin-8. Therefore, CD133 could be an “Achilles’ heel” for CSCs, because by inhibiting this protein, the signaling pathways that are involved in cell proliferation will also be inhibited (Figure 1) [44].

## 3. The Consensus Molecular Subtype Classification for CRC

In cancer, the establishment of tumor mass and its metastasis does not seem to depend only on the genetic and epigenetic charge of CSCs, but on the composition of TME. TME is commonly integrated into different cell types, cytokines, and extracellular matrix components. Researchers in 2015 proposed a new molecular classification of four consensus molecular subtypes (CMSs) to integrate these molecular and histologic features of CRC based on the transcriptomic sequences shown in Table 1 [45]. This classification has been used for prognostic value in metastatic rectal cancer [46].

The patient’s treatment scheme should consist of a combination of therapies, from first-line treatment, such as surgery, chemotherapy, radiotherapy, and secondary-targeted therapy. However, this depends on the cancer stage and its characteristics. Following this, transcriptome and genome analyses allow for a better stratification of CRC patients and selection of the appropriate immunotherapy strategy [47].

## 4. Cell Types Used in CRC Immunotherapy

### 4.1. T-Cells

T cells are part of the adaptive immune system, divided into CD4+ (helper) and CD8+ (cytotoxic) T cells. CD4+ T cells support the body´s adaptive response to different classes of pathogens by cytokine production. CD8+ cytotoxic T lymphocytes are activated in response to tumor-associated antigens present in the context of MHC class I molecules [52]. T cell immunotherapy has different strategies, such as increasing or inhibiting cellular immunity or inducing changes in T cell receptors to recognize specific targets.

Chimeric antigen receptor (CAR) T cell receptors are designed to (1) deliver strong activation, proliferation, and survival signals via a single binding event; (2) in an independent manner to MHC, bypassing the MHC downregulation by certain tumors; and (3) exhibiting a high affinity even at low antigen density. Techniques involving CAR T cell methods have evolved from the basic design, ectodomain antibody single-chain fragment, and variable fragment engineered to the T cell receptor (TCR)-chain to multiple CAR generations. As a result, CAR design has progressed from simple molecules to more complex moieties, allowing for the combinatorial antigen selection with diverse signaling properties. This advancement facilitates the development of armored CAR T cells with improved antitumor activity and good toxicity management.

#### 4.1.1. Checkpoint Inhibitor Drugs

T cell activity can be controlled by checkpoint inhibitor drugs that hinder the blocking of tumor-associated immunosuppression and allow cytotoxic cells and lymphocytes to attack tumor cells. However, in advanced CRC, immune checkpoint therapy is limited to patients with high microsatellite instability (MSI-H) (approximately 5%). This type of CRC is associated with high rates of tumor mutation (tumor mutational burden high—TMB-H) and tumor-infiltrating lymphocytes [52].

Tumor-infiltrating lymphocytes expressing Fas are very susceptible to Fas-mediated apoptosis. Thus, inhibition of FasL on colon cancer cells improves antitumor immunity and reduces tumor growth. However, serum levels of FasL increase in colon cancer and have a decreased or mutated expression of FasR, TRAIL-R1, and TRAIL-R2 death receptors on their cell surface, promoting survival [53].

#### 4.1.2. Adoptive T-Cell Therapy

Adoptive T cell therapy (ATC) takes patient-derived ex vivo expanded T cells and reinfuse them into patients [52]. Unlike conventional T lymphocytes that recognize peptide antigens bound to highly polymorphic MHC molecules, Vγ9Vδ2 T cells recognize nonpeptidic antigens without antigen processing and MHC restriction. Vγ9Vδ2 T cells stay preactivated, lacking antigen exposure. An in vivo administration of compounds (aminobisphosphonates and IL-12) that activate Vγ9Vδ2 T cells or an adoptive transfer of ex vivo expanded cells are necessary to drive its antitumor activity. In vitro, Vγ9Vδ2 T cells present strong cytotoxic activity against tumor cell lines or primary cells from colon carcinoma [53]. Moreover, γδ T cells are one of the most prominent immune cells in the gut and good candidates for immunotherapeutic strategies in CRC [52].

CSCs in CRC are commonly resistant to T cell therapy. As a form of sensibilization, chemotherapeutic drugs used for CRC treatment, such as 5-fluorouracil and doxorubicin, have been used in CSCs from CRC cell lines in combination with autologous Vγ9Vδ2 T cells, increasing DR5 expression and improving cytotoxic activity at low doses [53].

Adoptive cell transfer (ACT) is a form of cell therapy, in which T cells isolated from cancer patients’ tumors are modified by engineering, they are selected, expanded ex vivo, then reinfused into the patients [4]. ACT has already been clinically successful in treating hematologic malignancies; however, current evidence suggests that T cell therapy can improve treatment against CRC, even at advanced disease stages [54]. ACT therapy of ex vivo expanded αβ T cells (anti-CD3 and IL-2 stimulation) in combination with chemotherapy XELOX (capecitabine and oxaliplatin), and bevacizumab have achieved an 80% response rate and acceptable toxicity in stage IV CRC [55].

#### 4.1.3. T-Cell Receptor Therapy

In T cell receptor therapy (TCR), the T cell receptor is modified to target a specific antigen presented by an MHC molecule. In patients with CRC, carcinoembryonic antigen (CEA) is a frequently upregulated common target antigen [52]. As another strategy, transgenic TCR can bind with CEA+ CRC cells, enhancing tumor recognition compared to wild-type T cells [56] In a study of three patients with metastatic CRC, targeted therapy of TCR reduced CEA levels between 74–99%, and one patient had metastasis regression. However, severe transient inflammatory colitis toxicity occurred [57]. Clinical trial NCT02757391 is testing CD8+ T cell therapy in combination with pembrolizumab (PD-1 inhibitor) [52].

#### 4.1.4. Chimeric Antigen Receptor T Cell

The chimeric antigen receptor T cell or CAR-T cell is a genetically modified T cell that can avoid the MHC and directly target a surface antigen of interest. CAR receptors comprise a target-binding extracellular region that gives antigen specificity conformed by a single-chain variable fragment (scFv) antibody, a hinge and transmembrane region, and an intracellular domain related to T cell activation via the TCR CD3ζ signaling chain [58]. CAR-T cell therapy attempts to express functional chimeric receptors that recognize tumor antigens in a non-MHC-restricted manner, allowing for the recognition of any desired target. Despite its success in hematologic diseases, its response in solid tumors, such as CRC, is less than 9%, mainly due to the lack of uniformly expressed target antigens [52,59]. In solid tumors, such as CRC, surface proteins, such as the natural killer group 2, member D (NKG2D), and CEA are proposed [59].

The first clinical trial of CAR-T cells for CRC (NCT02349724) used CEA as a target. Treatment showed that 70% of patients with progressive disease and those previously treated presented stable disease for more than 30 weeks and a decreased tumor volume without severe secondary effects. Applying anti-CEA CAR-T cells in CEA+ adenocarcinoma with liver metastasis (NCT02416466 and NCT02850536) intraarterially administered, improved the delivery of cells into the metastasis and reduced cytokine release syndrome (CRS) [58]. However, before the employment of CAR-T cell therapy, several changes in the tumor microenvironment should be considered, as in a clinical trial of CEA CAR-T cells that increased the resistance of CEA+ rectal cancer tumor immunity [60].Two current phase I trials are recruiting subjects. The NCT04107142 trial uses CAR-T cells targeting NKG2DL, and NCT03970382 is testing neoantigen-targeted TCR on locally advanced or metastatic tumors. In patients, applying different quantities of autologous and allogenic NKG2D CAR-T cells showed that 1 × 10^8^ and 3 × 10^8^ cells were achieved without dose-limiting toxicity [59]. In NCT03692429 against NKG2D ligands, a modified TCR was used to make them suitable for allogeneic use with a TCR inhibitory molecule (TIM) sequence. Patients remained stable for approximately 3 months after treatment without graft vs. host disease [58]. Another antigen implicated in tumor growth and regulation of EMT in CRC is doublecortin-like kinase 1 (DCLK1). DCLK1-scFv (CBT-511) CAR-T cells induced cytotoxicity and increased IFN-γ release in coculture with CRC cells [52,59].

The human epidermal growth factor receptor 2 (HER2) is a potential target for CRC treatment, since it is highly overexpressed in this type of tumor. For metastatic CRC, HER2-targeted CAR-T cells eliminated numerous HER2+ solid tumors presenting signs of prevention of CRC progression in a xenograft model [54,61]. Guanylyl cyclase c (GUCY2C), a membrane-bound receptor overexpressed in more than 95% of CRC metastases, is a potential target. In a syngeneic murine model of CRC, GUCY2C CAR-T cells provided long-term protection against lung metastases [62]. However, independently of the high CAR-T cell efficiency, a problem with this therapy is the development of CRS, frequently observed by the overactivation of T cells. CRS can present simple fatigue or develop into life-threatening outcomes with a capillary leak through a severe increase of cytokines, such as IL-1, IFN-γ, and TNF-α [59].

### 4.2. Natural Killer (NK) Cells

Natural killer (NK) cells are lymphocytes that differ from the B and T cells belonging to the innate immune system. They originate in the bone marrow and are found in blood and lymphatic tissues, especially the spleen. Morphologically, they are large lymphocytes with cytoplasmic granules [63], and their characteristic phenotype is TCR-, BCR-, CD3-, CD16+, and CD56+. Its main functions are cytotoxicity and cytokine secretion [64]. According to the expression of CD56, they are classified into two subsets: the CD56 low/dark, which is antitumor cytotoxic, and CD56 bright [58,65] NK cells are a subset of immune effector cells that play an important role in immune activation against aberrant cells. Unlike T cell activation, NK cell activation is mediated by the contact of NK receptors with target cells. This mechanism is independent of antigen processing and presentation. An advantage of NK cells is their inherent ability to discriminate between healthy and malignant cells. NK cells express germ-line encoded activation and inhibitory receptors that trigger activation while balancing activating and inhibitory signaling. NK cells are activated by receptors that recognize stress-induced ligands on the surface of malignant cells. Normal cells express inhibitory receptors as self-major histocompatibility complex (MHC) class I molecules [66].

Another type of NK cells is cytokine-induced cells (CIK) [60,67], a heterogeneous population of NKT cells co-expressing CD3 and CD55 derived from T cell precursors. NK cell-mediated targeting and destruction of malignant cells is defined by an interplay of signals generated by inhibitory and activating NK cell receptors, that interact simultaneously with their ligands on target cells [68]. The ability of NK cells to destroy and eliminate target cells is determined by the balance between their activating and inhibitory signals, i.e., ligands expressed on target cells. These ligands interact with the NK cell surface receptors and trigger activating or inhibitory signals; thus, antigen specificity does not control NK cells [69]. In addition, the destruction of tumor cells by NK cells is not dependent on MHC or antibodies, these cells are attracted by the stress that characterizes TME [63]. Moreover, CSCs commonly express low or no MHC class I, which makes them susceptible to NK cell targeting. In some cases, CSCs also overexpress NK cell activating markers, such as CD24, CD44, CD133, and ALDH, facilitating its elimination by stimulating NK activation markers, such as MICA/B, Fas, and death receptors [70]. Thus, it is considered the ideal target because the expression of MHC-1 decreases, resulting in NK cell activation by a self-missing recognition process.

Various regulatory and stimulatory cytokines, such as IL-2, IL-21, IL-12, IL-8, IL-15, and interferon type 1, promote NK cell activities on tumor elimination [71]. Upon activation, NK cells release cytotoxic granules containing perforin and granzymes that directly lyse tumor cells, including activated cytotoxic T cells [72]. The NK cell receptor activator NKG2D, tumor necrosis factor (TNF)-related apoptosis-inducing ligand (TRAIL) [73], and perforin-mediated pathways [74] are important for CIK cell-mediated recognition and lysis of malignant cells.

In the history of NK cells as a treatment for neoplasms, a greater number of reports have been seen in acute megaloblastic leukemia [75], in which its effect has been evaluated by allogeneic transplantation of hematopoietic progenitors from haploidentical donors [76] in adult patients using NK lymphocytes from the donor. Focusing on the innate rather than the adaptive immune system, NK cells are not specific for antigens, such as T cells. They can bypass the engagement of tumor cell PD-L1 and T cell PD-1 to mediate direct cytotoxicity against CSCs [54]. For this reason, researchers are studying NK cells to potentiate their function in vitro before infusion in ACT as ex vivo allogeneic NK cells with a mixture of cytokines termed cytokine-induced memory-like NK cells (CIML-NK), which can combine with CAR engineering [77]. Based on the goal of inducing antigen-specific T cells in patients, using DC vaccines as antigen-presenting mechanisms that target tumor-derived blood vessels to disrupt tumor angiogenesis and decrease tumor growth is another form of immunotherapy for CRC patients [78,79]. NK cells can differentiate from induced pluripotent stem cells (iPSC). This source avoids the current requirements of collection and expansion as IPSCs can grow indefinitely by self-renewal. Furthermore, IPSC-derived NK cells are homogenous and clinically produced in a scalable manner [1]. Moreover, this approach allows for multiple genetic modifications to improve NK cell cytotoxicity [2]. There are diverse methods for genetic modification of iPSC-derived NK cells, such as lentivirus, transposons, and CRISPR-Cas9 system [3]. Thus, genetically engineered iPSC-derived NK cells could represent a promising strategy for a renewable source of NK cells for immunotherapy of solid tumors, such as CRC.

As other NK cell populations, iPSC-derived NK cells exhibit cytotoxic activity through the release of perforins and granzymes, the production of proinflammatory cytokines as interferon gamma (IFN-γ) and tumor necrosis factor alpha (TNFα), and apoptosis induction by TRAIL and Fas-FasL interaction [2]. iPSC-derived NK cells can uniformly express hnCD16, showing a potent antibody-dependent cellular cytotoxicity, a common mechanism of NK cells cytotoxicity [1]. In addition, iPSC-derived NK cells have been used in ovarian cancer models of NOD/SCID/γc^−/−^ (NSG) mice, improving survival from 73 to 98 days, indicating the therapeutic potential for treatment of solid tumors [4,5].

The NCT03841110 clinical trial used iPSC-derived NK cells for lymphoma and advanced solid tumor treatment, such as CRC. This treatment was combined with immune checkpoint inhibitors (nivolumab, pembrolizumab, or atezolizumab). It showed that 69% of solid tumor patients had the best response of stable disease without graft versus host disease (GvHD) or neurotoxicity (NT) [77].

The NK-92 cell line was originally established from a 50-year-old male patient with rapidly progressive non-Hodgkin lymphoma. It was selected due to its characteristics of activated NK cells, the lack of expression of inhibitory killer Ig-like receptors (iKIRs), lack of immunogenicity, and expansion facility. The safe use of NK-92 cells has been analyzed in phase I clinical trials [80]. Another study used NK-92 cells modified to target EpCAM among a CRC line. HCT-8-Luc cell line was implanted in a subcutaneous xenograft NOD/SCID mouse model, showing that CAR-NK-92 cells significantly reduced tumor growth. The use of combinations among other treatments and cell therapy is being explored for NK cells. The synergistic effects of regorafenib and CAR NK-92 cells, which recognize EpCAM+ CRC cells, and release cytokines, such as IFN-γ, perforin, and granzyme B, while reducing tumor xenografts in mice, were observed [80].

Two fully humanized single-chain DNA fragment variable antibodies recognizing CD16 on NK cells and CD133 on CSCs, were spliced, creating a novel drug defined by 16 × 133 novel bispecific killer cell engagers (BiKE). This molecule simultaneously recognizes antigens to facilitate an immunologic synapse. The 16 × 133 BiKE is a potent engager of the innate immune system capable of inducing NK cell degranulation and IFN-γ production and mediating selective targeting of CD133+ CSCs. The 16 × 133 BiKE may have therapeutic potential in a clinical NK cell therapy program for carcinomas, as it could serve as an alternative therapy for drug-resistant CSCs due to its unique mechanism of action [43].

### 4.3. Macrophages

Most macrophages are found in the gastrointestinal system, where they eliminate infections, control inflammatory reactions, preserve homeostasis, and regulate insulin sensitivity [81] Monocytes from the circulation are drawn to the tumor site by macrophages in response to environmental stimulation. They are polarized into tumor-associated macrophages (TAMs), the most prevalent immune cells in the TME of CRC. Exosomes, or the production of various cytokines, are two ways TAMs might interact with tumor cells to encourage their growth, invasion, migration, and angiogenesis. TAMs secrete the chemokine CCL2, that attracts regulatory T cells (Tregs), blocks T cells’ antitumor immunological responses, and disrupts immune cell connections, creating the immunosuppressive milieu of CRC [82,83]. Moreover, TAMs interact with the microbiota in CRC and use various metabolic pathways [84]. The TME is a special habitat that emerges when the tumor progresses [75]. The TME’s immune cells, particularly TAMs, are a significant part of the TME. They actively contribute to tumor formation, invasion, metastasis, immunosuppression, angiogenesis, and drug tolerance by secreting cytokines and chemokines and working with inflammatory processes [85,86].

#### 4.3.1. Preventing Monocyte Infiltration in CRC

A promising approach to treating initial tumors is to prevent mononuclear cells from infiltrating the inflammatory tissues connected to the tumor. The transcription factors HIF-1, CXCL-12, and CXCR4 are more highly expressed in the hypoxic TME environment, according to Chanmee T. et al. The HIF-1/CXCR4 pathway can be targeted to prevent TAM buildup [86,87]. Moreover, NT157 belongs to a new family of anticancer medications that inhibits tumor cells by targeting the STAT3 oncogenic signaling pathway and the IGF-1 receptor (IGF-1R). The expression of tumor-promoting cytokines, chemokines, and growth factors, such as IL-6, IL-11, and IL-23, CCL2, CCL5, CXCL7, CXCL5, intercellular adhesion molecule-1 (ICAM1), and TGF-, is inhibited by NT157, according to studies, which prevents TAMs.

#### 4.3.2. Repolarizing TAMs

TAMs can be re-educated by promoting polarization from the M2 to the M1 phenotype, since they mostly display the M2 phenotype and support angiogenesis and immunosuppression [84]. For instance, Georgoudaki et al. evaluated the impact of immune checkpoint therapy by repolarizing TAMs to the M1 type and inducing anticancer activity in a mouse model of MC38 colon cancer by suppressing the expression of the macrophage receptor with collagenous structure (MARCO) by TAMs [84,88]. Tasquinimod, small-molecule immunotherapy, alters the frequency and amount of tumor-infiltrating myeloid cells to lessen the TME’s immunosuppressive potential [89]. Tasquinimod induces phenotype switching from the proangiogenic and immunosuppressive M2-like phenotype to the pro-inflammatory M1-like phenotype, that changes the TME to promote immunomodulation, prevent angiogenesis, and inhibit metastasis, according to research by Olsson et al. [84,89]. Tasquinimod targets early-stage tumor-infiltrating myeloid cells. T2 RNases, which are evolutionarily conserved tumor suppressors, can slow tumor growth in vivo by bringing in adaptive antitumor CD8+ T lymphocytes and balancing the M1/M2 macrophage ratio in tumors [84,90]. Furthermore, Halama, N. and his colleagues also confirmed that inhibiting CCR5 can repolarize the phenotype of TAMs from M2 to M1 by regulating the STAT3/SOCS3 signaling pathway in TAMs, thereby exerting antitumor effects in a phase I clinical trial of patients with CRC liver metastases [84,91].

Korehisa et al. reported that in colon cancer patients with high microsatellite instability PD-L1 is mainly expressed by aggressive front-end tumor cells and CD68/CD163-positive M2 macrophages, and PD-L1 expression is associated with features, such as poor tumor differentiation, lymphatics, etc. Gordon and collaborators found that PD-1 expression by TAMs increased as the disease progressed. Further experiments showed that PD-1 expression negatively correlated with the phagocytic capacity of TAMs, and in vivo blocking of PD-1-PD-L1 increased the phagocytic capacity of macrophages, leading to tumor progression that was shown to decrease and prolong mouse survival [92,93].

### 4.4. Stem Cells

Mesenchymal stem cells (MSCs) can be modified to overexpress therapeutic proteins that inhibit tumor growth or activate apoptosis. Cytokines and interferons have been used as immunotherapy regulators against cancer [94,95]. Interferons can suppress tumor cell proliferation and alter the immune response [96]. Strategies that combine IFN with tumor-specific antibodies or standard chemotherapeutic medications, effectively inhibit cancer progression in animal models [97].

Several cytokines suppress tumor growth by the selective induction of apoptosis or by potentially infiltrating adaptive and innate immune cells [98]. Following this statement, bone marrow (BM)-MSCs highly express PAI-1, and in some colon cancer cell lines increase migration and proliferation, while in others, such as HCT-116, they decrease growth [99]. BM-MSCs are capable of homing more easily in the presence of CD133+/CD44+ cells than CD133-/CD44-; moreover, these markers are found in CSCs, and according to these results, colonic CSCs have a greater capacity to recruit BM-MSCs. BM-MSCs are in co-culture with CD133+/CD44+ cells, and the levels of IL-8 secreted by the MSCs increase, which is associated with liver metastasis [100]. Hombach, in 2020, reported an in vitro assay with MSCs modified by a retroviral vector to release both IL-7 and IL-12, shifted the chronic inflammatory profile in the tumor tissue into a more favorable one for an acute CAR-T cell response in CRC [101].

However, a xenograft model with MSC modified with IL-7/IL-12 increased the effectiveness of CAR-T cell therapy against colorectal cancer [102]. In addition, MSCs derived from human placenta (hP-MSC) modified and combined with appropriate transgenic therapeutic gene herpes simplex virus truncated thymidine kinase-HSV-ttk/prodrug (ganciclovir) produced highly effective cytotoxicity on colon cancer cells (HT-29) [103]. Thus, they may be used for treating tumors in vivo. As another immunogenic chemoattractant of NK cells, Th1 lymphocytes and macrophages highlight CXC3R1, which is upregulated in CCR and inflamed tissues, and participates in improving the trans-endothelial migration of MSCs. In particular, rat BM-MSC expressing CX3CR1 and IL-25 improved immunomodulatory activities in the colitis colon [104].

Several studies have shown that MSCs fully expressing TRAIL can induce apoptosis in colon cancer cell lines HCT-15 and DLD-1 [35]. However, one of the main limitations to its efficacy as a treatment is the development of resistance. Some of the main resistance mechanisms associated with the apoptotic effect mediated by TRAIL are (1) decreased DR4 and DR5 receptors, (2) expression of decoy receptors (DcR1 and DcR2), and (3) overexpression of antiapoptotic genes (cFLIP, Bcl-XL, and Bcl-2) [105]. Recent work has evaluated the use of chemotherapeutic agents to sensitize TRAIL-resistant cells by mediating the apoptotic effect of recombinant TRAIL, resulting in the proposal that chemotherapeutic agents, such as paclitaxel, could be used as a pre-treatment for sensitizing CD133+ (CSC marker) to the effect mediated by TRAILs by reducing the expression of the antiapoptotic genes cFLIP and Bcl-XL in pancreatic cancer [106]. Although TRAIL-resistant tumor cells exist, Mueller et al. (2011) proved that, in selected colon cancer cells, TRAIL-MSC could overcome resistance via direct intercellular interaction, thereby inhibiting the growth of HCT-8 and HT29 cells [107].

#### 4.4.1. MSC as a Platform for Suicide Gene Delivery

Modified MSC with an insert of a suicide gene carrier can activate a non-toxic pro-drug to become a cytotoxic substance capable of eliminating tumor cells. The key advantage of this technique is that it amplifies drug toxicity inside the tumor, resulting in the death of adjacent cells due to indirect effects generated by the transformed MSCs [108]. The active prodrug’s cytotoxic impact increases the release of toxic chemicals that activate immune cells, including cytotoxic T cells and macrophages, resulting in more efficient cancer destruction [109].

Some examples of pro-drugs are ganciclovir (GCV) of 5-fluorouracil (5-FU), which have been used with a herpes simplex virus thymidine kinase to produce toxic metabolites utilized in combination with MSC to target various malignancies [110]. Moreover, MSCs genetically modified to carry the HSV-TK suicide gene selectively affect tumor stroma, reducing primary tumor growth [111]. Among clinical trials, in patients with advanced gastrointestinal adenocarcinoma, MSC_apceth_101 treatment in combination with GCV demonstrated safety and tentative signs of effectiveness [112]. Adipose tissue-derived, MSC engineered to express yeast CD inhibited colon cancer growth when combined with 5-FU in an immunocompromised mouse model [113]. In mouse xenograft models, co-administration of CD expressing MSCs with 5-FU was also effective in treating melanoma and human prostate cancer. Furthermore, in the presence of GCV, TRAIL, and HSV-TK, modified MSCs greatly reduced tumor development and enhanced survival in mice models of highly aggressive glioblastoma multiforme (GBM) [114].

Another target evaluated was NK4, an intramolecular HGF fragment consisting of an N-terminal hairpin domain and four HGF-chain Kringle domains (K1–K4). This fragment binds to MET without triggering receptor signal transduction. It was reported that NK4 gene expression enhances 5-fluorouracil-induced apoptosis of murine colon cancer cells [95]. The systemic administration of MSC transduced with inhibitor NK4 suppressed the growth of gastric cancer xenografts and reduced intra-tumoral vascularization [96,115].

#### 4.4.2. MSC with an Oncolytic Virus (OV)

This therapeutic strategy is based on viruses and selectively targets replicating tumor cells, destroying them by cytolysis. Once the cell is lysed, it can release viral particles capable of infecting more neighboring tumor cells. An additional benefit of OVs is that the lytic nature of cell killing induces immunogenic cell death (ICD), increasing the recruitment of immune cells to the tumor site. The main OVs studied were adenovirus human serotype 5 (Ad5), coxsackievirus and adenovirus receptor (CAR), conditionally replicative adenovirus (CRAd), and ONYX-015, with a few pre-clinical and clinical trials in different types of cancer. MSCs carrying oncolytic adenovirus with gene-directed enzyme-prodrug therapy hold potential and provide superiority in cancer gene therapy. Recent studies have demonstrated that the combination of MSC-delivered OV with prodrug activation increases the efficacy and safety of CRC therapy. This strategy is a novel combination in which suicide gene therapy is based on delivering a foreign gene that encodes a prodrug-activating enzyme, followed by systemic administration of a non-toxic prodrug that is subsequently converted into a potent cell-killing drug [116].

## 5. Limitations of Cellular Therapy on Advanced CRC Treatment

**Figure 2:**
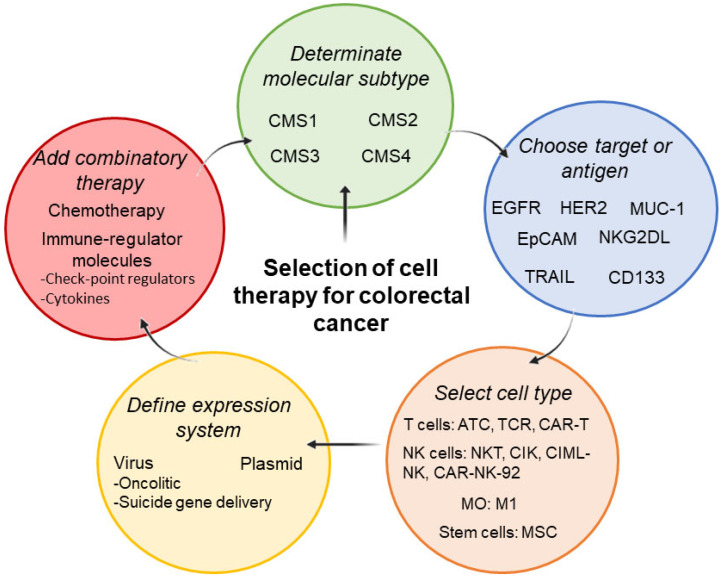
Schematic steps to consider for the selection of cell therapy as a treatment for CRC.

In this review, we discussed the advantages and status of the immune cell-based therapy to fight CRC. Current clinical trials of cell therapy in CRC are included in Table 2. In addition, in the Figure 2 we described the aspects that should be considered for selection in immunotherapy-based cell therapy and gene therapy as treatment against CRC. However, inherent disadvantages are addressing the tumoral tissue and the technical drawbacks implied in such therapies that may hinder their utilization in advanced CRC. All ACT therapies require conditioned/genetically modified cells to exert their anticancer effects. Tumor-infiltrating lymphocytes (TIL) have been proven effective in several types of cancer, such as melanoma [117], ovarian cancer [118], breast cancer [119], cervical cancer [120], and others. These therapies require tumor-adjacent T cells from the patient to expand tumor-conditioned cells in vitro and infuse them back into the patient [121].

CAR-T cells can be isolated from peripheral blood and genetically modified and expanded in-vitro [122]. Alternatively proposed options include a combination of treatments, local administration of cells and different CAR structures. Toxicity due to exacerbated immune reaction is a latent risk in immunotherapy, especially on CAR-T cells, since they can trigger a more potent reaction. CRS is a systemic inflammatory response [123] that can be initiated as a result of the T cell therapy and can be fatal (on-target toxicity) [124]; countermeasures are available, such as the administration of tocilizumab (anti-IL-6 receptor mAB) [58]; interestingly, CRS is dependent on tumor burden [60], which is inconvenient for advanced CRC, Therefore, CAR-T cell therapy is not recommended for these patients unless tumor reduction is achieved by other therapies [125,126]. An off-target effect can also occur as some antigens of the malignant cells can also be present in healthy cells, being potential targets [109,126].

Tumor mass recruits several immune cells (Treg, myeloid-derived suppressor cells, TAMs) to achieve immunosuppression and evasion by secreting anti-inflammatory cytokines, such as IL-10 or TGF-B that hinder T cell proliferation and overall performance [127]. However, 4th and 5th-generation CAR-T cells include expression of pro-inflammatory cytokines (IL-12 or IL-15) as a counter mechanism to tumoral immunosuppression [128].

Another mechanism to increase CAR-T therapy efficacy is the inhibition by CRISPR/Cas9 technology of the immune checkpoints, such as PD-1, CTLA-4, and LAG-3, as well as VIST (V-domain Ig suppressor of T cell activation) and TIGIT (T cell immunoreceptor with Ig and ITIM domains) [60]. Nevertheless, CAR-T cells might be designed to express CXCR3 and CCR5 in the membrane to release heparinase from TILs [58]. Moreover, an important risk of CRS could be reduced by administering a monoclonal antibody against the IL-6 receptor (tocilizumab) [58].

Normally, the effectiveness of CAR-T cell therapy on hematological cancer is because malignant cells are circulating and exposed to infused T cells; however, this has proven to be an obstacle on solid tumors, since they are normally poorly irrigated; moreover, the heterogeneous mass of tissue, fibers, and cell receptors keep most CAR-T cells to the external surface, as only 9% of cells can enter the tumor mass [129].

In addition, each infusion of TILs or CAR-T is finite, implying that each infusion is expected to harness its antitumor abilities properly and proliferate; however, tumor tissue is extremely aggressive to non-cancerous cells. Hypoxia [130], nutrient-starved [131], and the abundance of acidic by-products of the accelerated metabolism characterize tumor tissue [126]. T cells are heavily affected by such a hostile environment. T cells hardly exert several cell functions associated with hypofunction, cell exhaustion, and reduced efficacy, possibly resulting in treatment failure [132]. Procuring infusion of a higher T cell ratio can extend the effective cells; however, it may be inconvenient for advanced CRC as it may demand more time to manufacture [133].

Even with the high effectiveness of CAR-T cells for solid tumors, these cells present an uncontrolled activity against the target antigen. Other studies of HER-2 CAR-T cells in metastatic colon cancer showed adverse secondary effects, such as targeting normal lung tissue expressing basal levels of HER-2. This finding is important because of the high risk of developing CRS and neurotoxicity using CAT-T cell therapy [59].

Researchers are currently using MSCs in diverse ways to target CSCs and improve the effectiveness of other cellular therapies by enhancing their homing capacity and reducing the side effects of standard cancer treatments. To illustrate, MSCs may repair damage caused by cancer radiotherapy thanks to their self-regeneration potential, tumor-targeting capacity, and paracrine functions [117]; however, they can promote cell growth and tumor progression. Genetically modified MSCs may serve as a medium in enzyme/prodrug therapy to target CSCs by expressing a transgene for an enzyme to convert a non-toxic compound into a cytotoxic drug [134]. This is another way in which MSCs function as a novel drug delivery platform and eliminate or promote apoptosis as target cells [135].

Nevertheless, the lack of clinical trials hinders the application of MSC-based therapies while, according to the literature, MSCs are a double-edged sword in managing CRC (Table 3). Evidence shows that MSCs can inhibit tumoral cell proliferation, migration, and infiltration, preventing CRC occurrence and progression. However, under different conditions, MSCs induce an immunosuppressed microenvironment, impairing immunological sensitivity and promoting tumor growth and recurrence [136,137]. While stem cell therapy offers promising results in its research, these cellular therapies against colorectal CSCs have limited clinical experience, possible side effects, and mixed results. More studies are needed to evaluate the safety and efficacy of these therapies for CRC treatment and to determine the best methods for delivering stem cells to the tumor site. Other limitations are the high demand in processing time, technical equipment, and costs. Available treatment is crucial for advanced CRC to improve prognosis, and it must correlate with the cost of current therapies [138].

Lastly, it is relevant to note that a patient’s treatment plan will typically include a combination of therapies, such as surgery, chemotherapy, radiotherapy, and targeted therapy, which will depend on the stage and characteristics of each cancer. Therefore, it is necessary to evaluate which cell type is the best immunotherapy strategy for cell therapy, depending on the molecular subtype [139].

## 6. Perspectives

Until now, knowledge of clinical trials with cell therapy as targeted therapy is still limited and therefore it is not possible to predict the best or worst treatment for patients with colon cancer. For this reason, we consider that more research on this topic is still necessary to evaluate the best strategy and methods used depending on the type and stage of the tumor. In addition, given the characteristics of CSCs, it is necessary to continue developing techniques to generate new combinatorial strategies that make it possible to completely eradicate this type of malignant cell more efficiently, offering less aggressive treatments for health purposes. Cell therapy continues to be one of the main candidates for this, since, in addition to serving as a therapeutic agent against CSCs, they may be capable of acting as delivery systems for anticancer molecules, which in the future could improve the quality of life of cancer patients by reducing the aggressiveness of current treatments.

## Figures and Tables

**Table 1:** Colorectal cancer molecular subtypes.

Subtype	CMS1-MSI Immune Subtype	CMS2, the Canonical Subtype	CMS3, the Metabolic Subtype	CMS4, the Mesenchymal Subtype	Ref
Mutations	MSI is high along with CIMP+, BRAF, and a low prevalence of SNCA.	WNT, MYC, SCNA (high activation), and BRAF.	KRAS-activating mutations; moderate or low mixed state of MSI and intermediate CIMP; moderate activation of WNT and MYC, with PIK3CA mutation and IGBP3 overexpression.	TGFβ activation; EMT; High SCNA.	[47,48,49]
Immune characteristics	Low CD8 and CD4 T cells, and NK cells infiltration.	Lack of DC recruitment. Absent of immune infiltration and regulatory cytokines.	Poorly immunogenic without immune infiltrates or regulatory cytokines.	High infiltration of CTLs, macrophages, and stromal cells; overexpression of EMT markers, such as TGF-B and CXCL-12.	[45,50]
Frequency Predominance	Proximal colon (14%)	Distal colon and rectum (37%)	Without predominance (13%)	Distal colon and rectum (23%)	[51]
Prediction of immunotherapy response	Good response (80%); Poor response to immunotherapy (20%).	Only 18% of CMS2 patients.	Only 19% of CMS3 patients.	26% of CMS4 patients are not candidates.	[46]

**Table 2:** Clinical trials of cell therapy and colorectal cancer.

Title	Clinical Trial ID	Colorectal Cancer Stage	Trial Phase	Administration Route	Cell Type	Status
NKG2D CAR-T cells to treat patients with previously treated liver metastatic colorectal cancer.	NCT05248048	Metastatic	Early Phase I	Hepatic artery transfusion	CAR-T (NKG2D)	Recruiting
T cell receptor-based therapy of metastatic colorectal cancer.	NCT03431311	Advanced metastatic (MSI+)	I/II	Intravenous (i.v.) injections	T cells (TGFβRII)	Terminated
A single-arm pilot clinical study of chimeric antigen receptor T cells combined with interventional therapy in advanced liver malignancy.	NCT02959151	Metastatic	I/II	Vascular interventional therapy or intra-tumor injection	CAR-T cells (CEA)	Unknown
Clinical study of CEA-targeted CAR-T therapy for CEA-positive advanced malignant solid tumors.	NCT05415475	Advanced	I	Intravenous infusion or intraperitoneal injection	CAR-T cells (CEA)	Recruiting
A clinical study of CEA-targeted CAR-T cells in the treatment of CEA-positive advanced malignant solid tumors.	NCT05396300	Advanced	I	Intravenous infusion or intraperitoneal injection	CAR-T cells (CEA)	Recruiting
A single-arm pilot clinical study of chimeric antigen receptor T cells combined with interventional therapy in advanced liver malignancy.	NCT02959151	Metastatic	I/II	Vascular interventional therapy or intra-tumor injection	CAR-T cells (CEA)	Unknown
A clinical research of CAR T cells targeting CEA-positive cancer.	NCT02349724	Relapse or refractory	I	Intravenous infusion	CAR-T cells (CEA)	Unknown
Hepatic transarterial administrations of NKR-2 in patients with unresectable liver metastases from colorectal cancer (LINK).	NCT03370198	Metastatic	I	Hepatic transarterial administration	NKR-2 cells (NKG2D)	Active, not recruiting
Dose escalation and dose expansion phase I study to assess the safety and clinical activity of multiple doses of NKR-2 administered concurrently with FOLFOX in colorectal cancer with potentially resectable liver metastases (SHRINK).	NCT03310008	Metastatic	I	Infusion administered concurrently with standard chemotherapy	NKR-2 cells (NKG2D)	Active, not recruiting
NKG2D CAR-NK cell therapy in patients with refractory metastatic colorectal cancer.	NCT05213195	Metastatic	I	Intra-peritoneal infusion	CAR-NK (NKG2D)	Recruiting
High-activity natural killer immunotherapy for small metastases of colorectal cancer.	NCT03008499	Metastatic	I/II	Intravenous infusion	NK cells	Completed
CAR-pNK cell immunotherapy in MUC1 positive relapsed or refractory solid tumor.	NCT02839954	Relapse or refractory	I/II	Intravenous infusion	CAR-pNK cells (MUC-1)	Unknown
ACE1702 in subjects with advanced or metastatic HER2-expressing solid tumors.	NCT04319757	Advanced or metastatic	I	Intravenous infusion	NK cells (HER-2)	Recruiting

**Table 3:** Comparison of each cell therapy as a treatment for CRC.

Cellular Therapy	Advantages	Disadvantages
**T cell therapy**	It has already been clinically successful in treating hematologic malignancies. Tumor-infiltrating lymphocytes (TILs) and genetically modified T cells (TCRs and CARs) can elicit a cytotoxic response, causing the apoptosis of CSCs.	Limited by biological barriers to the tumor mass and the immunosuppressive tumor microenvironment, such as a high level of hypoxia, low concentrations of nutrients, and the high release of acid products. [58]. Expression of a heterogeneous pattern of tumor antigens produced the evasion of antigen-specific CAR-T cells [52,124].
**NK cell therapy**	The expression of ligands for natural cytotoxicity receptors can mediate direct CSC apoptosis. Cytokine-induced memory-like NK cells (CIML-NK) can be combined with CAR engineering.	Limited by the immunosuppressive nature of tumor microenvironment and CRC, as seen in T cell therapy disadvantages. Difficulty in ex vivo expansion [5,79]. Low efficacy [70].
**Macrophage therapy**	M1 macrophages can cause tumor cell apoptosis through phagocytosis, antibody-dependent cellular cytotoxicity (ADCC), the release of molecules including TNF-α and nitric oxide (NO), and recruitment of cytotoxic T cells.	M2 macrophages contribute to angiogenesis, EMT of tumor cells, and immunosuppression, promoting the metastasis of CRC.
**MSC therapy**	Inhibits aberrant crypt foci formation and tumor development when administered in the early phase of colorectal tumorigenesis in rat models. Secretes cytokines that inhibit proliferation and induce apoptosis of CRC cells. Supports other cellular therapies by enhancing their homing capacity and healing tissue damage after radiotherapy. MSC-derived exosomes suppress proliferation, migration, and invasion of CRC cells through paracrine and direct tumor cell contact.	Induces an immunosuppressed microenvironment, resulting in impaired immunological sensitivity and the promotion of tumor growth and recurrence. BM-MSCs promote tumor growth by inducing the EMT progression of CRC cells in vitro. Pro-apoptotic and pro-survival effects can be difficult to predict and control.

## Data Availability

Not applicable.
